# Distribution of Liver Disease in Bangladesh: A Cross-country Study

**DOI:** 10.5005/jp-journals-10018-1092

**Published:** 2014-01-22

**Authors:** Salimur Rahman, Mohammad Faroque Ahmed, Mohammad Jamshed Alam, chitta Ranjan Debnath, Mohammad Izazul Hoque, Muhammad Mahbub Hussain, AKM Shamsul Kabir, Mohammad Fazal Karim, Faiz Ahmed Khondokar, Ahmed Khondokar Mamun-Al-Mahtab, Mohammad Golam Masud, Mohammad Kutub uddin Mollick, Ahmed Lutful Moben, Sheikh Mohammad Noor-E-Alam, Provat Kumar Podder, Aloke Kumar Raha, Mohammad Abdur Rahim, Mohammad Harun or Rashid, KMJ Zaki, Sheikh Mohammad Fazle Akbar

**Affiliations:** 1 Department of Hepatology, Bangabandhu Sheikh Mujib Medical University, Dhaka, Bangladesh; 2 Department of Hepatology, Dhaka Medical College, Dhaka, Bangladesh; 3 Department of Hepatology, Shaheed Suhrawardy Medical College, Dhaka, Bangladesh; 4 Department of Hepatology, Mymensingh Medical College, Mymensingh, Bangladesh; 5 Department of Hepatology, Comilla Medical College, Dhaka, Bangladesh; 6 Department of Hepatology, Rangpur Medical College, Rangpur, Bangladesh; 7 Department of Medicine, Holy Family Red Crescent Medical College, Dhaka, Bangladesh; 8 Department of Hepatology, Sir Salimullah Medical College, Dhaka, Bangladesh; 9 Department of Hepatology, Sher-e-Bangla Medical College, Barisal, Bangladesh; 10 Department of Hepatology, Khulna Medical College, Khulna, Bangladesh; 11 Department of Hepatology, Chittagong Medical College, Chittagong, Bangladesh; 12 Medical Assistant Training School, Noakhali, Bangladesh; 13 Department of Hepatology, Rajshahi Medical College, Rajshahi, Bangladesh; 14 Department of Hepatology, Sylhet M.A.G. Osmani Medical College, Sylhet, Bangladesh; 15 Department of Medical Sciences, Toshiba General Hospital, Tokyo, Japan

**Keywords:** Bangladesh, Distribution pattern, Liver diseases.

## Abstract

**Introduction:**

‘Hepatology’, as an independent discipline of medical science, has recently been established in Bangladesh. The aim of this study was to formulate the distribution of pattern of liver diseases in this country.

**Materials and methods:**

In this retrospective study, data regarding patients of liver diseases from the seven different administrative divisions of Bangladesh between January 2012 and 2013 were compiled.

**Results:**

The study included 59,227 patients (age ranged 15-95 years). Majority of the patients were males (67.9%). Although all patients appeared at the department of hepatology, 13.2% were diagnosed with liver diseases, but a vast majority of patients (77.35%) were suffering from nonulcer dyspepsia or irritable bowel syndrome. Patients with liver diseases were mostly suffering from chronic liver diseases (CLDs) (37 -69%). Complication of CLD, like hepatic encephalopathy, was less frequent in regions with better healthcare system. Nonviral infections, like liver abscess and biliary ascarisis, were not uncommon. Acute hepatitis was another very common entity and contributed to approximately 20% cases.

**Conclusion:**

This study provides insight about heterogeneous distribution pattern of liver diseases in different regions of Bangladesh.

**How to cite this article:** Rahman S, Ahmed MF, Alam MJ, Debnath CR, Hoque MI, Hussain MM, Shamsul Kabir AKM, Karim MF, Khondokar FA, Mahtab MA, Masud MG, Mollick MKU, Moben AL, Noor-E-Alam SM, Podder PK, Raha AK, Rahim MA, Rashid MHO, Zaki KMJ, Akbar SMF. Distribution of Liver Disease in Bangladesh: A Cross-country Study. Euroasian J Hepato-Gastroenterol 2014;4(1):25-30.

## INTRODUCTION

After it is humble beginning in Bangladesh in the 1990s, the ‘Department of Hepatology’, as an independent discipline of medical science, has come a long way in this country. Independent hepatology departments have been functioning in all major medical colleges of the country, as well as in the only medical university of Bangladesh. Bangladesh is the first country in this region to introduce postgraduation course in hepatology.

Although Bangladesh has a more or less homogeneous population, it is one of the most densely populated countries of the world, accommodating over 160 million population in an area of approximately 1,44,000 square kilometers. The country is one of the fastest growing economies in the world and there is a rapid shift of the country’s economy from an agro-based to an industrialized one.^1^ There is also continuous shift of rural population to urban due to increasing urbanization and also resultant changes in our lifestyle, mode of transmission of communicable diseases and incidence of both communicable and noncommunicable diseases in the country. However, the growth and resultant demographic changes are nonuniform, meaning that despite our homogeneous population, our disease patterns are likely to vary from one region to another. This is also manifested in case distribution of liver diseases in different parts of Bangladesh.

It is estimated that Bangladesh harbors about 8 million chronic hepatitis B virus (HBV) and chronic hepatitis C virus (HCV)-infected people.^2,3^ Also, there is frequent outbreak of viral acute hepatitis those are caused by hepatitis A virus (HAV) and hepatitis E virus (HEV). Also, the numbers of patients with noncommunicable disease like nonalcoholic fatty liver diseases (NAFLDs) are on increasing trend. During the 1st annual meeting of South Asian Association for the Study of the Liver (SAASL), at Dhaka, Bangladesh in 2013, the prevalence of liver diseases at hepatology department of different medical instructions of Bangladesh was compiled.

## MATERIALS AND METHODS

This is a retrospective study. A group of hepatologists from the seven different administrative divisions of the country was designated to analyze records from January 2012 to January 2013 of seven major public medical college hospitals of the country in order to give an overview of the pattern of liver diseases presenting at their respective department. These are Mymensingh Medical College in Dhaka division, Chittagong Medical College in Chittagong division, MAG Osmany Medical College in Sylhet division, Rajshahi Medical College in Rajshahi division, Rangpur Medical College in Rangpur division, Sher-e-Bangla Medical College in Barisal division and Khulna Medical College in Khulna division. The data thus generated were presented at a Special Scientific Session at the Inaugural Conference of South Asian Association for the Study of the Liver in Dhaka, Bangladesh from 18 to 19 May, 2013. Data analyses were limited to available information only and thus a complete analyses of data of different etiological factors from all regions were not possible.

## RESULTS

The study included 59,227 patients presenting at the department of hepatology in different medical institutions of Bangladesh. Of them, 19,200 patients were from Rajshahi division and 525 were from Rangpur division. They were between 15 and 95 years of age. Individuals below 15 years of age were excluded. Majority of the patients (67.9%) were males.

All the patients presented to their respective hepatology department assuming that they were suffering from some form of liver diseases. However on further exploration, 13.2% of the patients were diagnosed with liver diseases. The diagnoses were made on the basis of clinical examination, biochemistry and imaging.

Patients who presented with liver diseases were mostly suffering from chronic liver diseases (CLDs), ranging from 37 to 69%. Complications of CLD, like hepatic encepha-lopathy (HE) was less frequent (2.6%) in regions like Dhaka division, where the healthcare system is comparatively improved compared with regions like Khulna division (13.6%). In case of chronic hepatitis (CH), NAFLD appears to be an emerging epidemic.

Nonviral infections, especially liver abscess, is also quite common in Bangladesh varying from 0.4 to 12%. Biliary ascarisis is another common entity among liver disease patients seen in 5 to 10% cases.

Acute hepatitis is very common and is encountered round the year. HEV is the leading cause as in most countries of the region, followed by HBV. HAV is also encountered in significant number of adult patients, while drug-induced liver injury (DILI) contributes to minority of cases.

## DISCUSSION

Male predominance in our study is not surprising. Males suffer more commonly from HBV related CLD, including cirrhosis of liver. This is more so in males over 40 years of age and the average age of our patients was 51.95 years. Similarly, hepatocellular carcinoma (HCC) is also a disease of male predominance and it has been reported that HBV accounts for 61.5% cases of HCC in Bangladesh.^4,5^ Another study has reported that the average presenting age of Bangladeshi HCC patients is 41.92 years.^4^

In the absence of a structured referral system in Bangladesh, patients tend to choose their specialists based on their own assumption of the diseases they are suffering from. An important aspect of the study is that majority of the patients (86.8%) attending department of hepatology had no liver diseases; although they thought that they were suffering from some form of liver diseases. If we look at their presenting symptoms, we see that these included anorexia, nausea, vomiting, fever, jaundice, weight loss, abdominal discomfort and/or distension, and leg swelling. Although some of these symptoms are related to liver diseases, in most cases, these are vague and may be associated with wide variety of diseases. In fact, a vast majority of 77.35% patients with nonulcer dyspepsia or irritable bowel syndrome attended the hepatology departments. This clearly points to the taboos prevailing in our society regarding liver diseases, where anyone suffering from any discomfort tends to assume that such symptoms are related to hepatic dysfunction. This also justifies the need to create mass awareness about liver diseases in Bangladesh. Otherwise this is not only draining our resources, posing constrains to our health delivery system, this also contributes to unnecessary direct and indirect financial losses incurred by our patients, not to mention about the mental agony and sufferings of the patients and their families.

**Table 1:** **Table 1:** Incidence of chronic liver disease in Bangladesh

*Division*			*Percentage (%)*	
Dhaka			37	
Chittagong			50	
Sylhet			22.8	
Rajshahi			69	
Barisal			38	
Khulna			39	

**Table 2:** **Table 2:** Etiology of chronic liver disease in Bangladesh

Sylhet division		HBV (76%)		HCV (4.1%)		—		ALD (0.8%)		WD (0.4%)	
Barisal division		HBV (30%)		HCV (5%)		NAFLD (3%)		—		—	

ALD: alchoholic liver disease, HBV: Hepatitis B virus, HCV: Hepatitis C virus, WD: Wilson’s disease

When we look at the incidence of CLD (Table 1), although it remains the main form of underlying liver disease in our study population, there is significant variation in the percentage of patients who had CLD. In case of Dhaka divi sion, 37% patients had CLD. The figure is similar to that of Barisal (38%) and Khulna (39%) divisions, both located in the Southern part of Bangladesh. However, in Sylhet division only 22.8% patients had CLD, while the figures are extremely high and stand at 50 and 69% respectively in case of Chittagong and Rajshahi divisions. Barisal and Khulna are actually neighboring divisions and therefore a similar percentage of CLD in these two regions is not surprising. However, their similarity in the percentage of CLD with Dhaka division is difficult to explain, as these two regions are separated from Dhaka division by a major river gap, that is the Ganges. It is interesting that Ganges is also the river gap that separates Dhaka division from Rajshahi division, where the percentage of CLD is highest. On the contrary, geographically Chittagong and Rajshahi divisions are the farthest apart, whereas Chittagong and Sylhet are neighboring divisions; but unlike in case of neighboring Barisal and Khulna divisions, in this later case, the resemblance of percentage is between the farthest divisions.

As expected, HBV is the leading cause of CLD in the country (Table 2). This is similar to previous report from Bangladesh, where HBV is attributed to 61.15% cases of cirrhosis of liver in this country.^6^ However, there is also variation in the percentage of HBV as the etiologic agent of HBV in different regions of the country. The figure is as high as 76.5% in Sylhet division and as low as 30% in Barisal division. HCV was the second commonest cause of CLD responsible for 4.1% in Sylhet and 5% in Barisal divisions, respectively. Unlike in case of HBV, the incidence of HCV among CLD patients is rather similar. This is also supportive of previous observation.^6^ This is however a very important observation, as this raises the need for nationwide study to identify the exact prevalence of HBV in Bangladesh, so that proper emphasis may be given in allocating efforts and resources to sustain HBV in Bangladesh. Other causes of CLD as identified in the study include NAFLD, alcoholic liver disease (ALD) and Wilson’s disease (WD).

An interesting, but expected observation is that the complications of CLD are low in regions where hepato-logy services are developed compared with where it is not. For example, HE is relatively low in Dhaka division (2.6%), where the healthcare system is at it is best, but high in Khulna division (13.6%), where hepatology department has just been established (Table 3). On the contrary, in Barisal division this figure is in between at 5%, reflecting the status of hepatology care in this region, where it is not as developed as in Dhaka, but much better compared with that available in Khulna division.

**Table 3:** **Table 3:** Hepatic encepholopathy among chronic liver disease in Bangladesh

*Division*		*Percentage*	
Dhaka		2.6	
Barisal		5	
Khulna		13.6	

**Table 4:** **Table 4:** Incidence of nonalcoholic fatty liver diseases in Bangladesh

*Division*		*Percentage*	
Dhaka		1.1	
Sylhet		5.45	
Barisal		5	
Khulna		7.6	

**Fig. 1:**
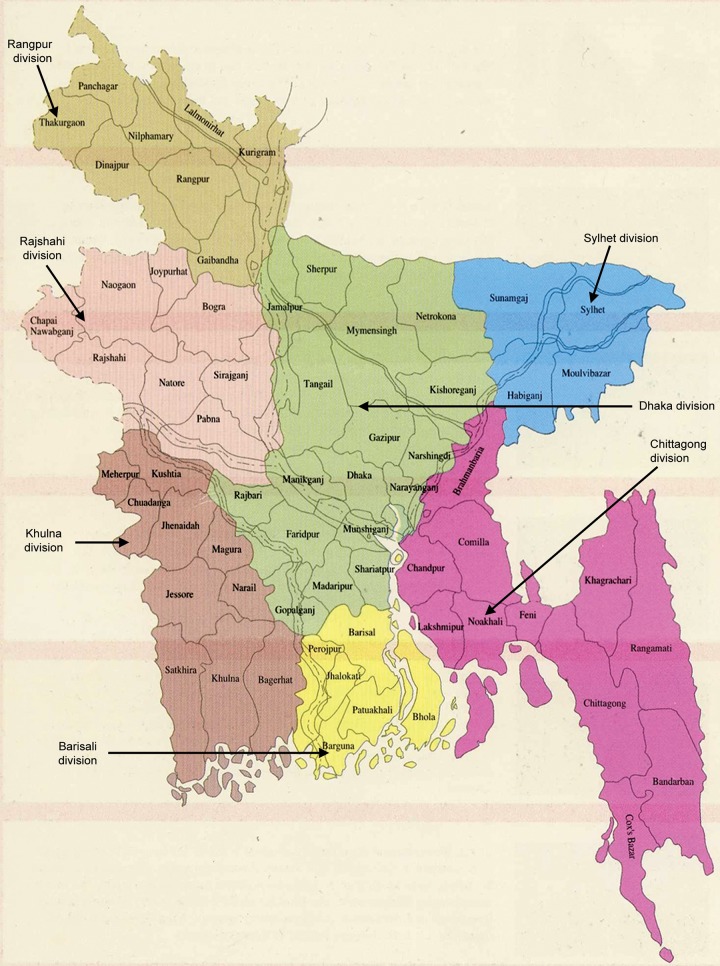
The administrative division of Bangladesh

**Table 5:** **Table 5:** Incidence of hepatocellular carcinoma in Bangladesh

*Division*		*Percentaoe (%)*	
Dhaka		8.4	
Chittagong		8	
Sylhet		0.6-1.36	
Rajshahi		2	
Barisal		5	
Khulna		4.8	

**Table 6:** **Table 6:** Incidence of liver abscess in Bangladesh

*Division*		*Percentaoe (%)*	
Dhaka		10	
Sylhet		0.4-0.6	
Rajshahi		5.2	
Barisal		10	
Khulna		12	

**Table 7:** **Table 7:** Incidence of biliary ascariasis in Bangladesh

*Division*		*Percentaoe (%)*	
Dhaka		8	
Chittagong		5	
Barisal		10	

**Table 8:** **Table 8:** Incidence of acute hepatitis in Bangladesh

*Division*		*Percentaoe (%)*	
Dhaka		27	
Chittagong		20	
Sylhet		11.36	
Rajshahi		11	
Rangpur		74.83	
Barisal		27	
Khulna		23	

However when we look at CH, NAFLD, appears to be an important etiology although the prevalence varying from as low as 1.1% to as high as 7.6% (Table 4). This is new information as previous study from Bangladesh^7^ has reported more patients with NAFLD. With Westernization of Bangladeshi society, it is not surprising that NAFLD is on the rise. Our neighboring India is considered as the ‘diabetes capital’ of the world and it has been predicted that with increasing obesity and metabolic syndrome in Asian Indian children, NAFLD is an emerging epidemic in our region as well as our country.^8^ The incidence of diabetes is also high in Bangladesh. With the absence of any definite ‘pharmacologic intervention’ for NAFLD yet, ‘lifestyle modification’ remains the key to management of NAFLD. It is high time that we create mass awareness about NAFLD.

In case of liver malignancies HCC leads the tally. However, there is marked variation in the incidence of HCC across different regions (Table 5). It is as low as 0.6 to 1.36% in Sylhet, but as high as 8 and 8.4% in Chittagong and Dhaka, respectively. The resemblance of incidence of HCC in neighboring Barisal (5%) and Khulna (4.8%) divisions is well understood, as the incidence of CLD is also similai in these two regions. The low incidence of HCC in Sylhe is also expected as this region also has the lowest incidence of CLD. Similarly not unexpected is the high incidence of HCC in Chittagong, which has large CLD population However, it is not clear why Dhaka division with a low prevalence of CLD has very high incidence of HCC and the opposite scenario is in Rajshahi, where the incidence of CLD is highest, but that of HCC is one of the lowest. As HBV is the commonest cause of both CLD and HCC in al**l** these regions a possible explanation is difficult to attain Factors like possible regional variation in HBV genotype food adulteration, and so on need to be investigated. Earlier studies have also found similar prevalence of HCC.^9^

The study also highlights other liver diseases encountered in our hepatology practice. Nonviral infections constitute significant percentage of our liver disease burden. The incidence of liver abscess is as high as 10 to 12% in some regions (Table 6). It is understandable that a relatively affluent region like Sylhet division will have a very low incidence of liver abscess (0.4-0.6%). It is however unclear why the capital region has similar incidence of liver abscess (10%) like Barisal (10%) and Khulna (12%) regions. Interestingly, like with CLD and HCC, Sylhet division enjoys the lowest incidence of liver abscess also, making it perhaps the ‘most liver friendly’ region of the country.

Another important noncommunicable liver disease is biliary ascariasis. Its percentage varying from 5 to 10% (Table 7). With better hygienic conditions, the contribution of biliary ascariasis to liver disease burden in Chittagong 5%. This is lower compared with Barisal division (10%), a coasta region that is criss-crossed by several rivers. This figure is in between (8%) in Dhaka division. Spurt of nonhomogeneous development with large number of slums still existing in the capital region may be the contributing factor.

Acute hepatitis is very common among our liver patients (Table 8). The figures are between 20 and 27% in most regions; percentages as low as 11% is encountered in some regions. HEV accounts for as high as 43.6% cases of acute hepatitis in Sylhet region followed by HBV being responsible for 38% cases there. HAV has been attributed to 10% cases in Barisal division, suggesting that this virus is becoming an important cause of acute hepatitis in our adul population. Improved sanitation and hygienic conditions are probably the contributing factors. Drug-induced liver injury, mainly with use of antitubercular drugs, is also reported in minority of the cases. However, detail data about the etiology of acute hepatitis are not available from most regions due to the relative unavailability of the investigations, cos involved and lack of definite therapeutic intervention foi most acute viral hepatitis. Such findings are similar to earlier observations from Bangladesh.^10^

## CONCLUSION

There is paucity of information regarding data on liver diseases, as well as other diseases, in Bangladesh. The study shows that although liver disease pattern is similar across Bangladesh, there is much variation in their distribution and prevalence. The study concludes that most liver diseases in this country are preventable. HBV is a major contributor to our liver disease burden, but can be prevented by early screening, successful vaccination, health education and awareness. Diabetes also poses significant threat being a major contributor to the upcoming NAFLD epidemic. Awareness also needs to be raised in this area.

If we are able to raise awareness among our people about liver diseases and ventilate how to block their transmission, we can expect to reduce the incidence and prevalence of liver diseases in Bangladesh. Emphasis should also be given on our National Health Policy on research in this field. At the same time, continuous scrutiny and data collection are needed for proper management of liver diseases.
